# Uninephrectomized High-Fat-Fed Nicotinamide-Streptozotocin-Induced Diabetic Rats: A Model for the Investigation of Diabetic Nephropathy in Type 2 Diabetes

**DOI:** 10.1155/2016/8317850

**Published:** 2016-12-20

**Authors:** Valentina K. Bayrasheva, Alina Yu. Babenko, Vladimir A. Dobronravov, Yuri V. Dmitriev, Svetlana G. Chefu, Ivan Yu. Pchelin, Alexandra N. Ivanova, Alekber A. Bairamov, Nina P. Alexeyeva, Ivan S. Shatalov, Elena N. Grineva

**Affiliations:** ^1^Institute of Endocrinology, Federal Almazov North-West Medical Research Centre, Saint Petersburg, Russia; ^2^Department for Pathophysiology, Pavlov First Saint Petersburg State Medical University, Saint Petersburg, Russia; ^3^Research Institute of Nephrology, Pavlov First Saint Petersburg State Medical University, Saint Petersburg, Russia; ^4^Institute of Experimental Medicine, Federal Almazov North-West Medical Research Centre, Saint Petersburg, Russia; ^5^Saint Petersburg State University, Saint Petersburg, Russia; ^6^Komarov Botanical Institute of the Russian Academy of Sciences, Saint Petersburg, Russia; ^7^Department of Statistical Modelling, Mathematics and Mechanics Faculty, Saint Petersburg State University, Saint Petersburg, Russia; ^8^Saint Petersburg National Research University of Information, Technologies, Mechanics and Optics, Saint Petersburg, Russia

## Abstract

Type 2 diabetes (DM2) could be reproduced in rats with alimentary obesity by using low doses of streptozotocin (LD-STZ) as well as STZ in high doses with preliminary nicotinamide (NA) administration. However, STZ could induce tubulotoxicity.* Aim*. To develop rat model of DN in NA-STZ-induced DM2 and compare it with LD-STZ-model in order to choose the most relevant approach for reproducing renal glomerular and tubular morphofunctional diabetic changes. Starting at 3 weeks after uninephrectomy, adult male Wistar rats were fed five-week high-fat diet and then received intraperitoneally either LD-STZ (40 mg/kg) or NA (230 mg/kg) followed by STZ (65 mg/kg). Control uninephrectomized vehicle-injected rats received normal chow. At weeks 10, 20, and 30 (the end of the study), metabolic parameters, creatinine clearance, albuminuria, and urinary tubular injury markers (NGAL, KIM-1) were evaluated as well as renal ultrastructural and light microscopic changes at weeks 20 and 30. NA-STZ-group showed higher reproducibility and stability of metabolic parameters. By week 10, in NA-STZ-group NGAL level was significantly lower compared to LD-STZ-group. By week 30, diabetic groups showed early features of DN. However, morphofunctional changes in NA-STZ-group appeared to be more pronounced than those in STZ-group despite lower levels of KIM-1 and NGAL. We proposed a new rat model of DM2 with DN characterized by stable metabolic disorders, typical renal lesions, and lower levels of tubular injury markers as compared to LD-STZ-induced diabetes.

## 1. Introduction

Appropriate experimental animal models of diabetic nephropathy (DN) are essential for studying its pathogenesis and different strategies of nephroprotection. The development of DN in type 2 diabetes (DM2) in most cases is triggered not only by hyperglycemia but also by other pathogenic factors associated with obesity, insulin resistance, hypertension, and dyslipidemia [1, 2]. In order to extrapolate relevantly preclinical data into clinical reality, animal models of DN in DM2 have to be based on the functional and structural lesion of human DN as well as metabolic abnormalities [3, 4]. It is especially valuable to reproduce accurately early diabetic changes in kidneys that are potentially reversible by pharmacologic interventions [5].

Nongenetic DN in DM2 is usually reproduced in rat models with varying degrees of streptozotocin-induced *β*-cell failure [6, 7]. *β*-cell-toxicity of streptozotocin (STZ) is related to its glucose-like chemical structure permitting STZ binding to GLUT 2 transporters expressed on *β*-cells [6, 8, 9]. STZ induces DNA fragmentation due to its alkylating activity [6–10]. The subsequent hyperactivation of DNA repair enzyme poly(ADP-ribose)polymerase (PARP1) has been shown to result in *β*-cell necrosis involving NAD^+^/ATP depletion [9].

Nicotinamide (NA), generally known as a predecessor of NAD+, is one of the PARP-inhibitors that could moderately attenuate STZ-induced *β*-cell damage and severity of DM [11–13]. Although STZ-NA-induced DM2 in rats was originally described in 1998 [13], models of DN induced by administration of NA and STZ in uninephrectomized overweight insulin-resistant rats have not been reported before.

Obesity and insulin resistance in outbred rats can be induced by high-fat feeding [14, 15]. Thus, most of described nongenetic rat models of DM2 are usually created by using low single or multiple doses of STZ in combination with high-fat diet of different composition and duration [16–20]. Some authors also use unilateral nephrectomy, which is considered to accelerate the progression of renal injury [6, 16]. As a consequence, according to available data, low-dose-STZ-injected high-fat-fed rats with or without unilateral kidney removal develop moderate hyperglycemia, obesity, insulin resistance, modest hypertension, hyperlipidemia, and moderate albuminuria [16, 19]. However, tubulointerstitial damage has not been evaluated in these models. At the same time, growing body of evidence indicates that the renal tubulointerstitium plays an important role in the onset and progression of DN [4, 21].

It is necessary to point out that renal tubular epithelial cells also express GLUT 2 transporter that makes them susceptible to STZ [8, 10, 22, 23]. Indeed, STZ belongs to a group of chemicals with established nephrotoxic ability [10]. Therefore, STZ usage might impose certain limitations on the interpretation of laboratory tests and renal morphology [6, 8]. This is especially important in case of DN modelling, because the presence of tubulointerstitial fibrosis is one of the hallmarks that must be used to validate animal models of DN. In 1995 Kraynak et al. showed that high-dose-STZ-induced DNA damage in renal tubular epithelial cell is transient, requiring up to 3 weeks for complete reparation [8]. However, it has remained unclear whether low doses of STZ could induce tubular injury and have long-term effects on renal changes in experimental DM2. As a consequence, it is still unknown whether NA as an established attenuator of STZ-mediated *β*-cell-toxicity could exert similar effects with regard to tubulotoxicity [11].

Recently established markers of tubular injury, neutrophil gelatinase-associated lipocalin (NGAL) and kidney injury molecule-1 (KIM-1), have been shown to detect toxic damage even before the presence of morphological changes [24–26]. Renal expression of KIM-1 and NGAL correlates with the extent of tubulointerstitial fibrosis and decline of renal function in both clinical and experimental settings [27]. Meanwhile, urinary KIM-1 and NGAL are elevated in type 2 diabetic rats with early signs of DN [28, 29], even not STZ-induced [28]. These findings are congruent with the results of clinical studies indicating that urinary KIM-1 and NGAL are sensitive biomarkers of early, preclinical stage of DN in patients with DM2 [30–32].

In our study, we assessed early and long-term effects of low-dose STZ injection compared to NA-STZ administration on renal disturbances in heminephrectomized high-fat-fed rats in order to optimize experimental modelling of STZ-induced DM 2 and DN.

## 2. Materials and Methods

### 2.1. Animal and Experimental Design

**Figure 1:**
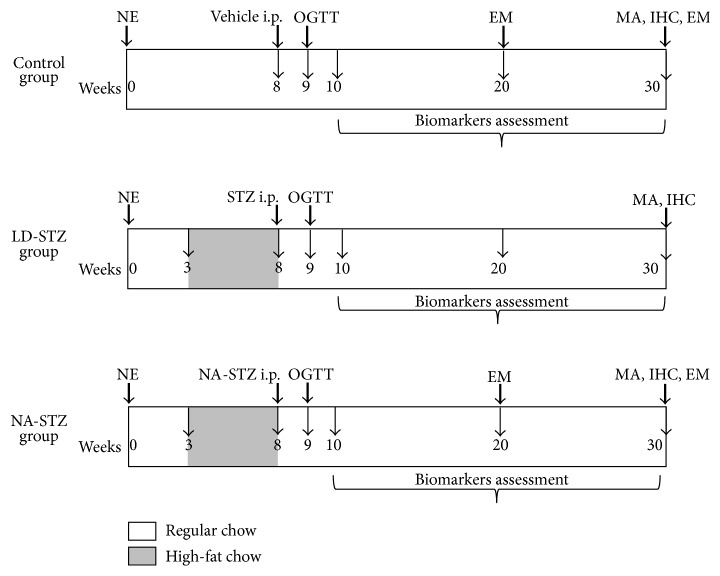
Schematic diagram of the experimental protocol. NE: right-side nephrectomy; STZ: streptozotocin; NA: nicotinamide; MA: light microscopy and morphometric analysis; IHC: immunohistochemistry; EM: electron microscopy; i.p.: intraperitoneally; OGTT: oral glucose tolerance test. NA-STZ group: uninephrectomized rats received the high-fat diet containing beef tallow for 5 weeks (from week 3 till week 8), and then successive intraperitoneal injections of NA (230 mg/kg) and STZ (65 mg/kg) in 15 min interval were administered. LD-STZ group: uninephrectomized rats received the same high-fat diet at the same period of the experiment, and then intraperitoneal injection of STZ in a lower dose (40 mg/kg) was administered. Control group: uninephrectomized rats received the regular commercial rodent chow during the experiment and received vehicle instead of STZ/NA-STZ.

Figure 1 shows the experimental protocol of the study. All experiments were performed in accordance with the “Guide for the Care and Use of Laboratory Animals” (publication no. [NIH] 85-23). All animal procedures were approved by the local ethics committee.

Thirty 8-9-week-old male Wistar albino rats weighing 180 to 210 g each were housed in 12-hour light/dark altered room at a constant temperature of 24°C, with food and water available* ad libitum*. All rats were subjected to right nephrectomy under anesthesia with sodium pentobarbital (50 mg/kg intraperitoneally) and then randomly divided into three experimental groups (10 animals in each group).

Three weeks after the surgery, two groups of experimental animals started the high-fat diet with finely chopped beef tallow (subcutaneous part, which contained 94% of fats, 1.5% of proteins, and 4% of water) mixed with coarsely chopped regular commercial chow for the next five weeks. The energy content of the diet was 450 kkal per 100 g (20% from fats, 48% from carbohydrates, and 20% from proteins). The measured range of food consumption per day was 30–40 g for one animal. All rats in both groups were transferred to regular chow diet after week 8 and then after overnight fasting were injected intraperitoneally by either

1. STZ in a low dose (40 mg/kg - LD-STZ group) or
2. NA in a dose of 230 mg/kg and a high dose of STZ (65 mg/kg) with a 15-minute interval (NA-STZ group);
3. 10 control uninephrectomized nondiabetic rats throughout the experiment were fed the regular commercial rodent chow containing 310 kkal per 100 g (5% from fats, 48% from carbohydrates, and 18% from proteins). Eight weeks after the surgery, control rats were intraperitoneally injected by vehicle (0.5 mL of sodium citrate buffer). The measured range of food consumption per day was 20–25 g for one animal.

Oral glucose tolerance tests (OGTTs) were performed weekly after injections of STZ/NA-STZ/vehicle.

After overnight fasting of rats at weeks 10 and 20, blood samples were collected via the tail vein. At the same time and at the end of the study (at week 30), rats were placed in metabolic cages, and urine samples were collected for 24 hours. Serum was separated by centrifugation at 3000 ×g for 10 min.

At week 20, two rats from the control and NA-STZ groups were anticipatorily sacrificed for kidney electron microscopic examination. At week 30, overnight-fasted rats were sacrificed; their kidneys were removed and processed for the light and electron microscopic examinations. At the time of sacrifice, blood was also collected from the aorta, and serum was separated as described above.

#### 2.1.1. Surgical Procedure of Right-Side Nephrectomy

**Figure 2:**
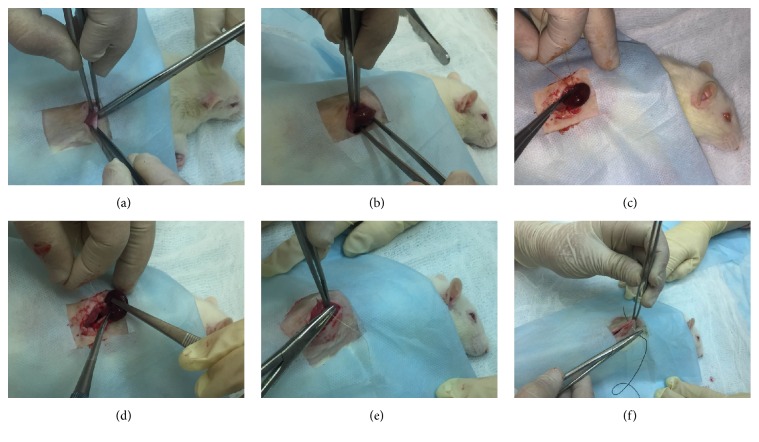
Surgical procedure of right-side nephrectomy. (a) After shaving under anesthesia, the right posterior and lateral abdominal wall is cleansed with skin antiseptic solution. Right vertical extra-peritoneal lumbar incision is performed. (b) Ligation of the right renal pedicle by 4/0 silk suture at two points. (c) Performing circular incision of renal capsule to prevent adrenal gland against extraction. (d) Separate enucleation and removing of the kidney. (e) Closing of the incision with simple continuous suture. (f) Closing of the skin with 2-0 silk simple intermittent sutures.

Figures 2(a)–2(f) and video file 1 (in Supplementary Material available online at http://dx.doi.org/10.1155/2016/8317850) show the surgical procedure of right nephrectomy. All of the operations were performed in sterile conditions in groups of rats between 8  a.m. and 12  p.m. for standardization. During one week after the surgery, rats were housed 2 per cage to protect against the seam discrepancy due to active movement and bellicose behavior of animals.

### 2.2. Materials

Streptozotocin, citric acid monohydrate, and trisodium citrate dihydrate were purchased from Sigma Aldrich (USA). Nicotinamide was purchased from Herba Hemosan (Austria). Primary rabbit polyclonal anti-collagen IV antibodies, secondary goat polyclonal antibodies HRP, and other reagents for IHC were purchased from Abcam (Great Britain). Kits for PAS-staining and Masson trichrome staining were purchased from Bio-Optica (Italy).

#### 2.2.1. Preparation of Citrate Buffer and Solutions of Streptozotocin and Nicotinamide

The solutions were prepared under sterile conditions using the laminar flow hood and then filtered through a 0.22 *μ*m filter (Millipore, USA).

All manipulations with STZ were carried out in the opaque laboratory flask, protected against the light by using aluminum foil. STZ powder, stored in dry, dark location at −20°C, dissolved in fresh prepared citric acid-sodium citrate buffer with pH of 4.5.

The buffer was prepared by mixing of 47 mL of 0.1 M citric acid solution (containing 21.01 g/L of citric acid monohydrate) with 53 mL of 0.1 M sodium citrate solution (containing 29.41 g/L of trisodium citrate dihydrate). Prepared solution was accurately mixed with distilled water up to the volume of 1000 mL, along with synchronous measurement of pH.

In case of the final pH exceeding 4.5, several drops of 0.1 M solution of citric acid were added. In case of pH less than 4.5, several drops of 0.1 M solution of sodium citrate were added.

NA was dissolved in 0.9% sodium chloride solution.

### 2.3. Determination of Metabolic Parameters

During the first ten weeks of the experiment, body weight was measured every 2-3 weeks. The fasting blood glucose levels were measured at the beginning of the high-fat diet, after the end of it, and during glucose tolerance test at week 9. From the beginning of week 10, body weight was estimated together with the fasting blood glucose level every 5 weeks.

One week after STZ/NA-STZ/vehicle injections, 40% solution of glucose (3 g/kg) was gently administered orally via a polyethylene gastric tube to at least 8 hours-fasted rats. Blood glucose levels were determined from the tail vein with a glucometer OneTouch Ultra, Johnson and Johnson (USA) at 0, 30, 60, 90, and 120 minutes. Rats with fasting blood glucose levels above 7.0 but less than 14.0 mmol/L, demonstrating at least 2-fold increase in calculated mean glucose area under the curve (AUC) or higher, continued the study [33]. Otherwise, rats were excluded from the study.

Total cholesterol, triglycerides levels were measured using commercial kits Cobas Integra, Roche (Germany), on clinical chemistry analyzer Cobas Integra 400 plus (Germany), Hemoglobin A1c, using commercial kit BioRad (USA) on HPLC-analyzer BioRad d10 (USA).

Serum insulin level was assayed by using rat insulin ELISA kit from ALPCO Diagnostics (USA) on ELx800 Absorbance microplate reader (USA). Insulin resistance was determined by Homeostasis Model Assessment (HOMA-IR). HOMA-IR was calculated according to Solskov et al. [34] using multiplying coefficient 174 to convert insulin in “ng/mL” units to “pmol/L”:

(1) HOMA-IR=fasting glucose (mmol/L)×fasting insulin (pmol/L)155.

### 2.4. Determination of Routine Renal Function Markers

Serum creatinine level, as well as creatinine level in collected urine samples, and serum urea level were assayed enzymatically by using commercial kits Cobas Integra, Roche (Germany), on clinical chemistry analyzer Cobas Integra 400 plus. Creatinine clearance (mL/min/kg) was calculated as

(2) $$\begin{array}{c} Creatinine\;\;clearance=\frac{24\text{-}hour\;\;urine\;\;volume\left(mL\right)\times urinary\;\;creatinine\left(mmol/L\right)}{1400\left(min\right)\times \left(Serum\;\;creatinine\left(\mu mol/L\right)\times 0\text{,}001\right)/rat\;\;weight\left(kg\right)}. \end{array}$$

Albuminuria was assayed by using rat albumin ELISA kit, purchased from Assaypro (USA), on ELx800 absorbance microplate reader (USA), and then total (24-hour) urinary excretion of albumin was calculated.

### 2.5. Determination of Renal Tubular Injury Markers

Urinary excretion of KIM-1 and NGAL was assayed by using rat ELISA-kits for KIM-1 and NGAL from Abcam (Great Britain) on ELx800 absorbance microplate reader. 24-hour excretion of these markers was calculated.

### 2.6. Glomerular Basement Membrane Evaluation by Electron Microscopy

To investigate the renal ultrastructure the segments of renal cortex tissue from anesthetized animals were fixed by immersion in 2.5% glutaraldehyde in 0.1 M cacodylate buffer with pH 7.2 for 12 h and postfixed in 1.0% osmium tetroxide for 1 h, dehydrated in a graded ethanol and acetone series, and embedded in Epon resin (EMbed 812, EMS, USA). Semithin and ultrathin sections were cut on ultramicrotome Leica EM UC7 (Austria). Semithin sections were stained with Epoxy Tissue Stain (EMS, USA), containing toluidine blue and basic fuchsine. Ultrathin sections were mounted on formvar coated grids and double stained with 0.5% uranyl acetate and 3% lead citrate. The sections were examined using transmission electron microscope Libra 120 plus from Carl Zeiss Microscopy GmbH (Germany), operating at 120 kV. Images were taken with a BM-2k-120 dual-speed on axis SSCCD camera from TRS (Germany). The GBM thickness was measured directly on the images by ImageJ software version 1.5 (National Institute of Health, USA). For this purpose, after calibration, the straight-line perpendicular from internal to external layer of GBM was applied in at least 30 chosen points (each 1 *μ*m) in all digital microscopic images. All basement membrane width measurements were expressed in nanometers, and the results were added to the statistic box.

### 2.7. Histological and Immunohistochemical Analysis

At week 30 of the study, experimental animals were anesthetized by intraperitoneal injection of sodium pentobarbital, and left kidney was extracted for histological examination. All kidneys after weighting and cutting in a half lengthwise were fixed with 4% paraformaldehyde for 24 hours and then embedded in paraffin. Semithin sections were stained by periodic acid-Schiff (PAS) reaction and Masson trichrome. The same investigator blindly evaluated all sections, and at least 35 glomeruli were analyzed in each rat kidney slide in inverted microscope Leica DMI6000 (Austria).

#### 2.7.1. Glomerular Injury Assessment

Glomerular injury was evaluated by using mesangial expansion and glomerulosclerosis indices in sections stained with PAS reagent and by analyzing the intensity of immunohistochemical staining for collagen type IV in glomerular region. A semiquantitative morphometric analysis was used to evaluate the degree of glomerular injury. Glomeruli were accurately graded in a sequential manner for avoiding mistakes related to grading the same glomerulus several times.

*Glomerular Mesangial Expansion Index Assessment*. Mesangial matrix expansion was defined by the presence of increased amounts of PAS-positive material in the mesangial region with or without hypercellularity. Thus, the degree of glomerular mesangial expansion was assessed using semiquantitative scoring method, where grade 0 means normal glomerulus; grade 1 means mesangial expansion area up to 25%; grade 2 shows 25–50% expanded mesangium; grade 3—50–75%; grade 4 means almost total mesangial expansion—75−100% [35]. In this way, glomerular mesangial expansion index for 1 rat kidney slide was calculated as follows:

(3) $$\begin{array}{c} \frac{\left(1\times N\;\;with\text{ “}1\text{”}\;\;score\right)+\left(2\times N\;\;with\text{ “}2\text{”}\;\;score\right)+\left(3\times N\;\;with\text{ “}3\text{”}\;\;score\right)+\left(4\times N\;with\text{ “}4\text{”}\;\;score\right)}{total\;\;amount\;\;of\;\;examined\;\;glomeruli}, \end{array}$$

were* N* is number of glomeruli.

Average score was calculated for each rat. Afterwards, we estimated mean values of these scores for each experimental group.

*Glomerulosclerosis Index Assessment*. Glomerulosclerosis index was calculated in the same semiquantitative manner [36]. Glomerulosclerosis was defined as the collapse or obliteration of the glomerular capillary tuft, or both, accompanied by arterial hyalinosis. Briefly, each glomerulus was graded from 0 to 4 as follows: grade 0, no lesions; grade 1, up to 25% involvement of the glomerular tuft with initial mesangial expansion and basement membrane irregularity; grade 2, lesion in 25% to 50% of glomerulus with mild segmental hyalinosis; grade 3, diffuse glomerular hyalinosis/sclerosis occupying 50% to 75% of glomerulus; and grade 4, meaning almost whole (more than 75%) of the glomerular tuft is obliterated.

*Immunohistochemical Staining for Collagen Type IV*. For immunohistochemical staining, 4 *μ*m thick-sections were placed on polylysine-coated slides (Menzel Glaser, Germany), and after overnight drying, the paraffin was removed by xylene. Then sections were placed in a graded ethanol series and immersed in distilled water. After heat-induced antigen unmasking, sections were immersed in 3% hydrogen peroxide solution for 20 min. To avoid nonspecific staining, the sections were then incubated in blocking solution for 1 hour at room temperature, followed by using primary rabbit polyclonal anti-collagen IV antibodies in dilution 1 : 500 overnight at 5°C. Then secondary goat polyclonal antibodies conjugated to HRP were added. At least 35 glomeruli of each kidney section were evaluated under ×100 magnification and classified semiquantitatively depending on the brownish yellow pigmentation intensity of glomerular basement membranes and mesangial matrix, as described by Liu et al. [37]. Briefly, each observed glomerulus was graded and coded as follows: 0, less than 25% staining; 1, 25–50% positive staining; 2, 50–75% positive staining; and 3, more than 75% positive staining. Average score was calculated for each rat. Afterwards, we estimated mean values of these scores for each experimental group.

#### 2.7.2. Tubulointerstitial Fibrosis Assessment

For image analysis of tubulointerstitial fibrosis, Masson trichrome and collagen type IV-stained sections were used. From each kidney, a total of 8 interstitial images of each slide (renal cortex and juxtamedullary region) were captured and processed with digital camera Leica DFC495 (8 Megapixel CCD) integrating into Leica DMI6000 inverted microscope (Austria) using the 10x objective. The area occupied by collagen fibers was measured using image analysis software ImageJ version 1.5 (National Institute of Health, USA) and classified semiquantitatively according to Bai et al. [38], where grade 0 indicated absence of changes; grade 1, fibrosis less than 25%; and grade 2, fibrosis occupying 25–50%; a score of 3 was arbitrarily assigned when approximately 50% or more of analyzed field was involved.

### 2.8. Statistical Analysis

Data were evaluated using the IBM SPSS Statistics version 22.0. All data are presented as “mean ± standard deviation.” Differences between the groups were analyzed by one-way ANOVA with* post hoc* Tukey's multiple comparison test in combination with the Mann–Whitney Rank Sum test when appropriate. The paired sample Wilcoxon signed-rank test was used to compare the results from available pairs of the same group at different time of measurement. Results of OGTTs were compared using one-way ANOVA with repeated measures, and Tukey's test was performed. Significance level was accepted at *P* < 0.05.

## 3. Results

### 3.1. Determination of Metabolic Parameters

#### 3.1.1. Changes of Body Weight

**Figure 3:**
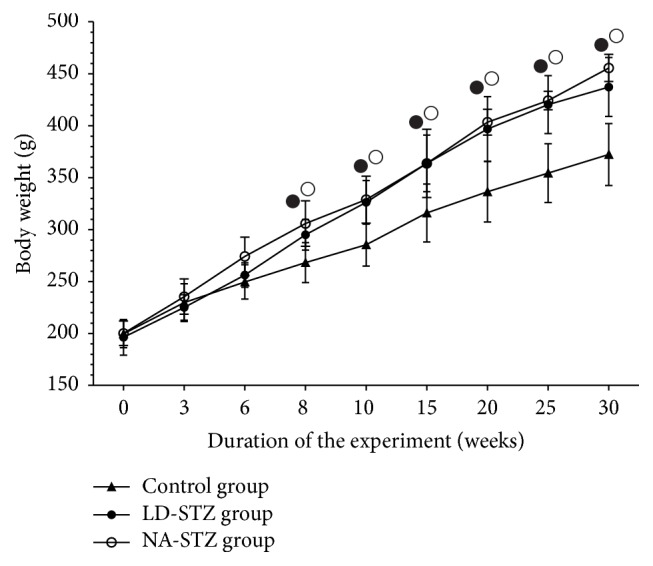
Changes in body weight (g) of rats in the experimental groups throughout the study. Dynamics of body weight starting at week 0 when unilateral nephrectomy was performed. LD-STZ and NA-STZ groups were fed with the diet containing beef tallow from week 3 till week 8 of the study. Mean values and standard deviations are shown. ○ – *P* < 0.05 in NA-STZ group versus control nondiabetic group; ● - *P* < 0.05 in LD-STZ group versus control nondiabetic group. Nonsignificant difference between NA-STZ and LD-STZ groups is not shown.

According to the data shown in Figure 3, the body weight gain during the study was not statistically significantly different between two experimental diabetic groups. Body weight of these animals started to increase 3 weeks after initiation of the high-fat diet and became significantly higher compared to control rats at week 8 of the experiment. It remained elevated throughout the follow-up period despite the regular chow resumption. Duration of high-fat feeding did not exceed 5 weeks, because the rats reluctantly consumed the chow with beef tallow starting at the fourth week of the diet. Rats completely refused to eat the high-fat chow at fifth week, necessitating its change to the regular chow. Body weight has become higher in NA-STZ group as compared to LD-STZ group since week 20, but these differences were nonsignificant.

#### 3.1.2. Stability of Experimental Diabetes Parameters

**Figure 4:**
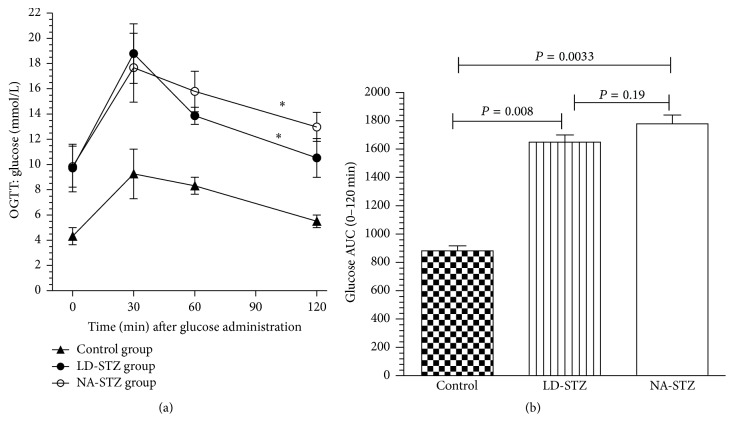
Glucose curves of oral glucose tolerance tests (OGTTs) and area under the curves (AUC) of glucose in the experimental groups. (a) Plasma glucose responses to oral administration of glucose in dose of 3 g/kg during 120 min OGTTs in the control (nondiabetic rats) and groups with diabetes being induced one week earlier (at week 8 of the experiment) by intraperitoneal injections of either a low dose of streptozotocin (LD-STZ) or nicotinamide and streptozotocin (NA-STZ). ^*∗*^ *P* < 0.01 compared to control nondiabetic group. Nonsignificant difference between NA-STZ and LD-STZ groups is not shown. (b) Total glucose area under the curves (AUC) is presented for comparison between experimental groups. Mean values and standard deviations are shown.

According to the results of OGTTs, single low dose of STZ (40 mg/kg) induced DM 2 in 40% of high-fat-fed rats and the combination of NA and a high dose of STZ (65 mg/kg) in 70%. As shown in Figure 4(a), all of the groups were characterized by the highest glucose level at 30 minutes of OGTT with gradual decrease in the following 90 minutes. However, diabetic rats demonstrated significantly higher fasting glucose levels as well as impaired glucose tolerance during 2-hour period of the test. Interestingly, the glucose level in NA-STZ group decreased slower throughout last 90 minutes of OGTT compared to LD-STZ group. Nevertheless, these differences were nonsignificant. The area under the curve (mmol/L × min) was not different in the two diabetic groups, but in both of them it was significantly higher than the same parameter in the control group (Figure 4(b)).

**Figure 5:**
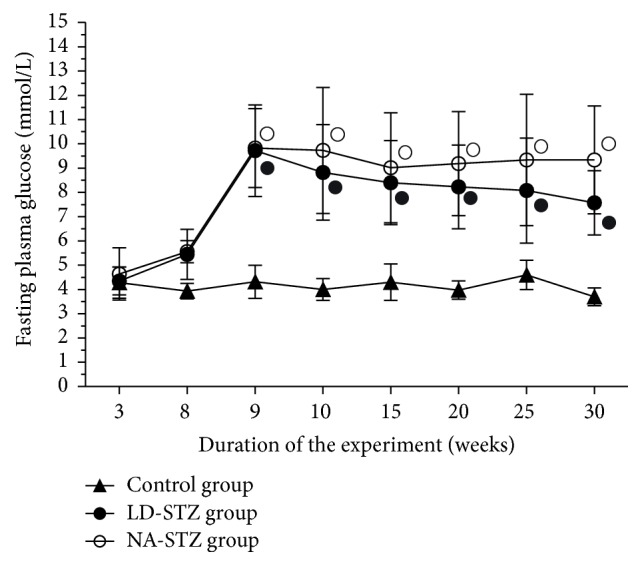
Fasting plasma glucose levels in the experimental groups. Overnight fasting blood glucose was measured for the first time 3 weeks after unilateral nephrectomy; the second point of measurement assessed the effects of five-week high-fat diet with beef tallow. OGTT was performed 1 week after STZ administration (at week 9). 2 weeks after induction of diabetes fasting blood glucose levels were measured every 5 weeks until the end of the experiment at week 30. Mean values and standard deviations are shown. ○ – *P* < 0.05 in NA-STZ diabetic group versus control nondiabetic group. ● - *P* < 0.05 in LD-STZ diabetic group versus control nondiabetic group. Nonsignificant difference between NA-STZ and LD-STZ groups is not shown.

The fasting blood glucose levels during the follow-up period are shown in Figure 5. The high-fat feeding did not significantly alter the fasting glucose level. All animals with verified mild hyperglycemia in both groups remained diabetic until the end of the experiment. This fact was proven by almost 2-fold increase in HbA1c concentration in comparison with the control group (Table 1). However, mean values of fasting glucose in LD-STZ-injected diabetic group were lower compared to NA-STZ group with a tendency to normalization by the end of the study (7.6 ± 1.33 mmol/L versus 9.3 ± 2.22 mmol/L, resp., *P* = 0.17). These results are consistent with nonsignificant higher level of serum insulin in LD-STZ group as compared to NA-STZ group (Table 1). As a consequence, calculated indices of insulin resistance (HOMA-IR) were significantly higher in NA-STZ-diabetic group compared to nondiabetic rats. HOMA-IR in LD-STZ group was not significantly different with the same parameter in the control group. However, the difference in HOMA-IR between the two diabetic groups was relatively small until the end of the study.

#### 3.1.3. Serum Lipids Profile

Under the influence of 5-week diet with beef tallow, cholesterol and triglycerides were simultaneously elevated in both diabetic groups with more pronounced degree of dyslipidemia in NA-STZ group. They were significantly higher compared to the control group at all points of measurement (Table 1). By week 30, the difference in total cholesterol level between both diabetic groups has been a tendency to become significant (*P* = 0.11).

### 3.2. Determination of Routine Renal Function Markers

**Figure 6:**
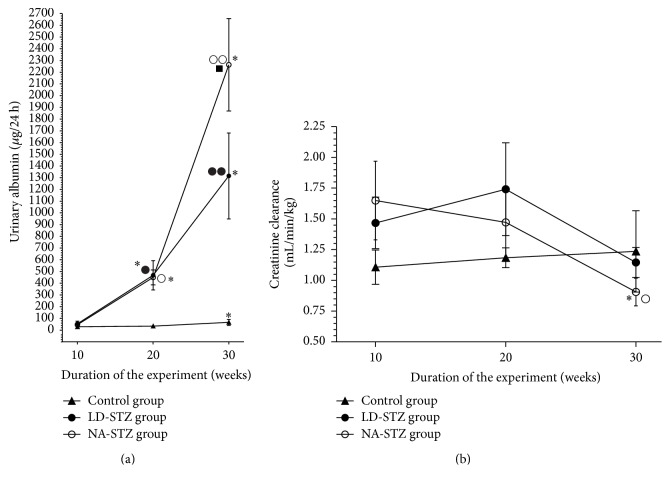
Routine renal function markers in the control, NA-STZ, and LD-STZ groups during the experiment. (a) Dynamics of urinary albumin excretion (*μ*g/24 h) measured at weeks 10, 20, and 30 of the experiment in the studied groups. (b) Dynamics of calculated clearance of creatinine (mL/min/kg) measured at weeks 10, 20, and 30 of the experiment in the studied groups. Mean values and standard deviations are shown. ○ – *P* < 0.05, ○○ – *P* < 0.005 in NA-STZ diabetic group versus control nondiabetic group at the same point of measurement. ● – *P* < 0.05, ●● – *P* < 0.01 in LD-STZ diabetic group versus control nondiabetic group at the same point of measurement. ■-*P* < 0.05 in NA-STZ group versus LD-STZ group at the same point of measurement. ^*∗*^ *P* < 0.05 compared to the first measurement in the same group at week 10.

As shown in Figure 6(a), neither LD-STZ nor NA-STZ groups demonstrated proteinuric levels of albuminuria by the end of the experiment. Nevertheless, since week 20 almost 10-fold higher urinary albumin excretion rate has been observed in both diabetic groups compared to control animals. Moreover, this distinction became stronger by week 30, when significant difference in urinary albumin excretion was identified even between both diabetic groups (2263.8 ± 394.5 *μ*g/24 h in NA-STZ versus 1315.6 ± 317.3 *μ*g/24 h in LD-STZ, *P* = 0.037), and albuminuria in NA-STZ group was more than 30-fold higher compared to control nondiabetic rats (71.4 ± 26.4 *μ*g/24 h, *P* = 0.004).

NA-STZ group showed a significant gradual decrease in creatinine clearance from the first measurement (1.57 ± 0.37 mL/min/kg) until the end of the study to 0.89 ± 0.10 mL/min/kg, *P* = 0.047 (Figure 6(b)). In contrast, creatinine clearance in LD-STZ-injected rats tended to have higher values at week 20 compared to NA-STZ group. However, significant difference was not observed at the end of the study. Creatinine clearance in nondiabetic group had a tendency to the hyperfiltration state presumably as a consequence of heminephrectomy [39].

### 3.3. Determination of Renal Tubular Injury Markers

**Figure 7:**
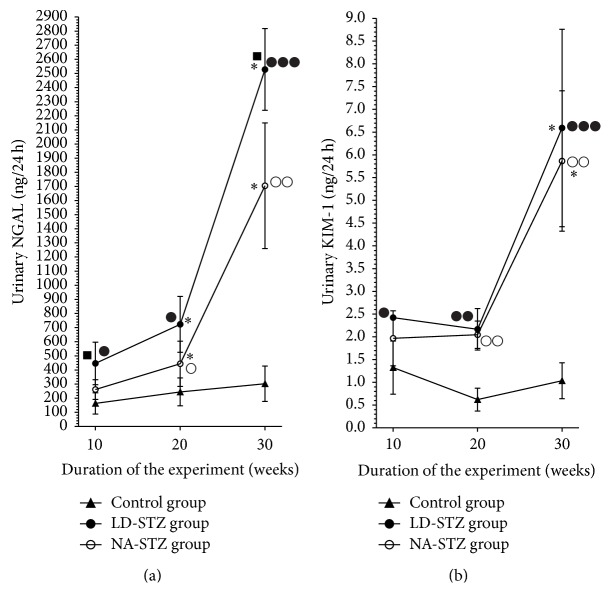
Dynamics of renal tubular injury markers in control, NA-STZ, and LD-STZ groups throughout the experiment. (a) Changes in urinary excretion of NGAL (ng/24 h) measured at 10, 20, and 30 weeks of the experiment in the studied groups. (b) Changes in urinary excretion of KIM-1 (ng/24 h) measured at 10, 20, and 30 weeks of the experiment in the studied groups. Mean values and standard deviations are shown. ○ – *P* < 0.05, ○○ – *P* < 0.01 in NA-STZ diabetic group versus control nondiabetic group at the same point of measurement. ● – *P* < 0.05, ●● – *P* < 0.01, and ●●● – *P* < 0.005 in LD-STZ diabetic group versus control nondiabetic group at the same point of measurement. ■-*P* < 0.05 in LD-STZ group versus NA-STZ group at the same point of measurement. ^*∗*^ *P* < 0.05 compared to the first measurement in the same group at week 10.

Urinary NGAL and KIM-1 levels did not significantly change during the study in control nondiabetic group (Figures 7(a) and 7(b), resp.). In contrast, we found several times increased values of both markers in diabetic groups by the end of the experiment compared to the initial state. Comparison with the control group showed significantly higher levels of NGAL and KIM-1 in LD-STZ group at all points of measurements. Noteworthy, there were no significant difference in these markers at week 10 in NA-STZ group compared to control rats (urinary NGAL: 261.2 ± 69.7 ng/24 h versus 172.65 ± 77.8 ng/24 h, resp., *P* = 0.19). At the same time, we obtained almost 2-fold increase of NGAL level in LD-STZ group compared to NA-STZ group (446.4 ± 150.2 ng/24 h, resp., *P* = 0.03). Moreover, that difference has remained significant by week 30 (2535.8 ± 303.9 ng/24 h in LD-STZ group versus 1704.4 ± 444.7 ng/24 h in NA-STZ group, *P* = 0.037).

### 3.4. Glomerular Basement Membrane Thickness Evaluation

**Figure 8:**
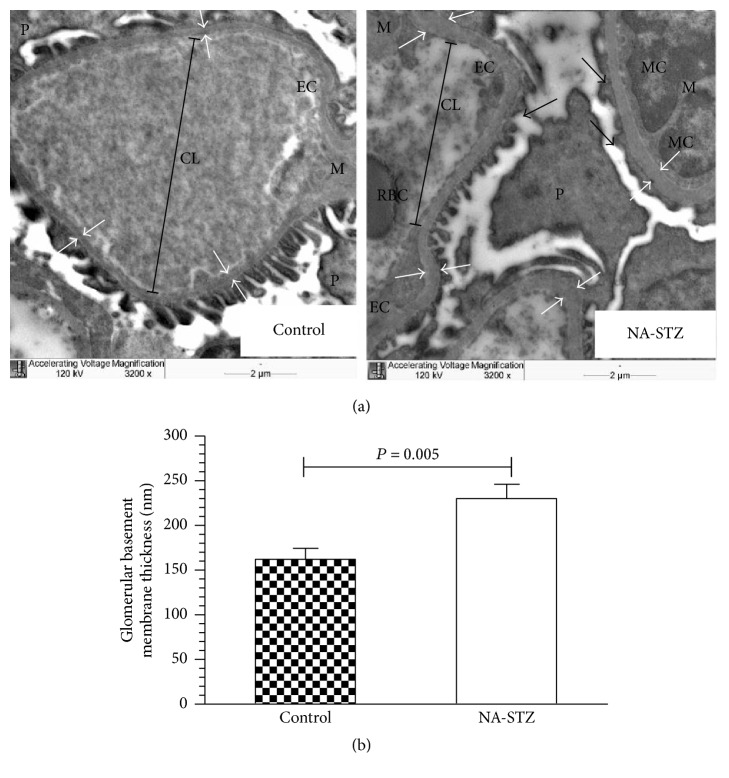
Transmission electron microscopic examination of the glomerular basement membranes (GBM) in control and NA-STZ-induced diabetic groups at week 20. (a) Representative electron microphotographs (magnification ×3200): white arrows indicate the thickness of the GBM; black arrows show foot process effacement characterized by loss of normal interdigitation pattern. Foot processes of the podocytes are fewer and wider; M: mesangium; EC: glomerular endothelial cell; MC: mesangial cells. Red blood cell (RBC) is seen in the capillary lumen (CL). Nicotinamide-streptozotocin-induced diabetes at week 20 caused early electron features of diabetic nephropathy such as marked irregular thickening of the capillary GBM and partial fusion of the podocytes in high-fat-fed heminephrectomized rats. The GBM of control rats is markedly thinner and no electron dense deposits are present in mesangial region. The mesangium from control nondiabetic rats is not prominent and does not compress the capillary lumen. (b) Quantification of the thickness of the GBM from control nondiabetic and NA-STZ-induced diabetic groups. Mean values and standard deviations are shown.

**Figure 9:**
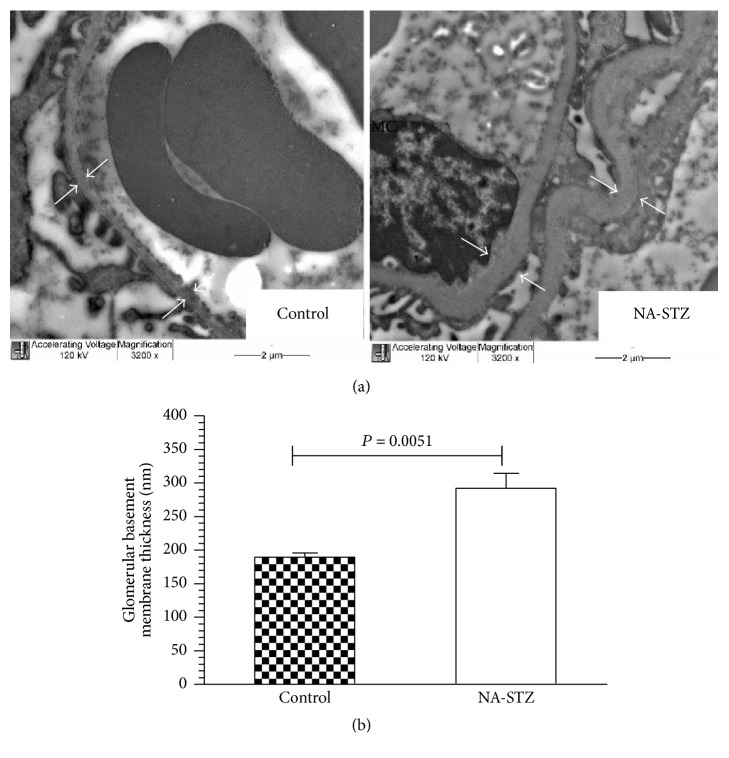
The results of transmission electron microscopic examination in control and NA-STZ-induced diabetic groups at week 30. (a) Representative electron microphotographs (magnification ×3200): white arrows indicate the thickness of the GBM. (b) Quantification of the thickness of the GBM from control nondiabetic and NA-STZ-induced diabetic groups. Mean values and standard deviations are shown.

As shown in Figures 8(a) and 8(b), the glomerular basement membrane (GBM) width, evaluated at week 20 of the experiment, was significantly increased in NA-STZ diabetic group (230.3 ± 38.5 nm) compared to control rats (170.7 ± 21.3 nm, *P* = 0.0049). This difference became even more evident by week 30 (299.1 ± 54.1 nm versus 189.7 ± 14.8 nm, resp., *P* = 0.0051) (Figure 9(b)). Electron microscopic evaluation was not performed in LD-STZ diabetic group at week 20 because of the critically small amount of animals who continued the study (4 rats). At the end of the experiment when the semithin sections of fragments from LD-STZ group were prepared, only juxtamedullary region was observed while glomeruli were not found.

### 3.5. Histological and Immunohistochemical Analysis

**Figure 10:**
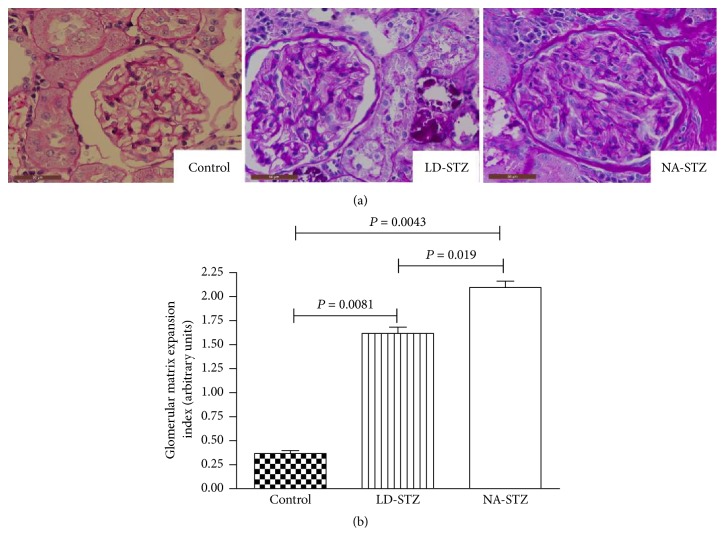
Periodic acid-Schiff (PAS) staining of renal glomerular regions from control and diabetic rats at the end of the experiment at week 30. (a) Representative images of PAS-stained kidney specimens from all experimental groups (magnification ×640). Glomerulus from control nondiabetic rat has opened capillary lumens and the fewest amounts of mesangial cells per capillary tuft. Moderate diffuse expansion of mesangium is observed in both diabetic groups. NA-STZ-injected diabetic rat shows markedly more expanded mesangium and endocapillary hypercellularity. (b) Graphic representation of semiquantitative indices of glomerular matrix expansion that, as indicated, are determined from at least 35 glomeruli in each kidney. Means and standard deviations are shown.

Semiquantitative glomerular matrix expansion index was significantly higher in both diabetic groups compared to nondiabetic animals. However, NA-STZ group showed significantly expanded mesangial matrix compared to LD-STZ group (Figure 10). It is necessary to point out that other light microscopic features of DN except tubulointerstitial fibrosis were more pronounced in NA-STZ group as well.

**Figure 11:**
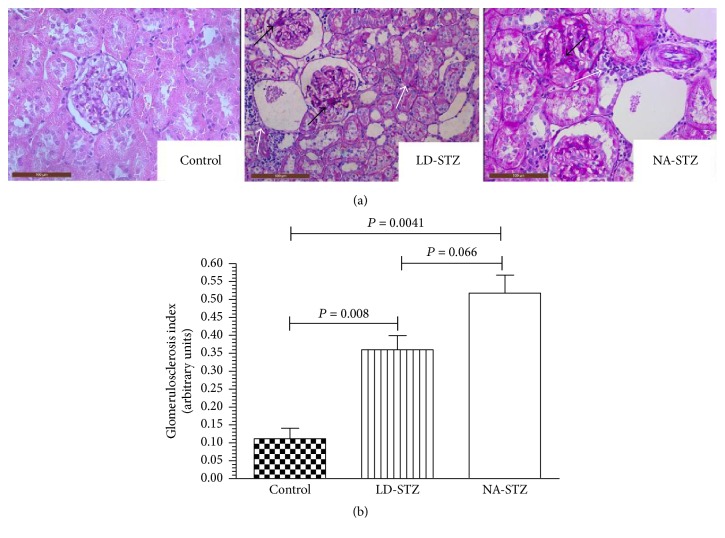
Glomerulosclerotic lesions in experimental groups at week 30. (a) Kidney sections were stained with periodic acid-Schiff reagent (magnification ×400). Representative microphotographs from two groups of diabetic rats show appearance of focal mesangial lesion with an acellular hyaline cores indicated by black arrows. Moreover, white arrows show interstitial inflammatory infiltration. Glomerulus from low-dose streptozotocin-induced diabetic rat demonstrates partial mesangiolysis and appearance of “lobular” structure of glomerular tuft. (b) Graphic representation of semiquantitative indices of glomerulosclerosis in the studied groups. Mean values and standard deviations are shown.

**Figure 12:**
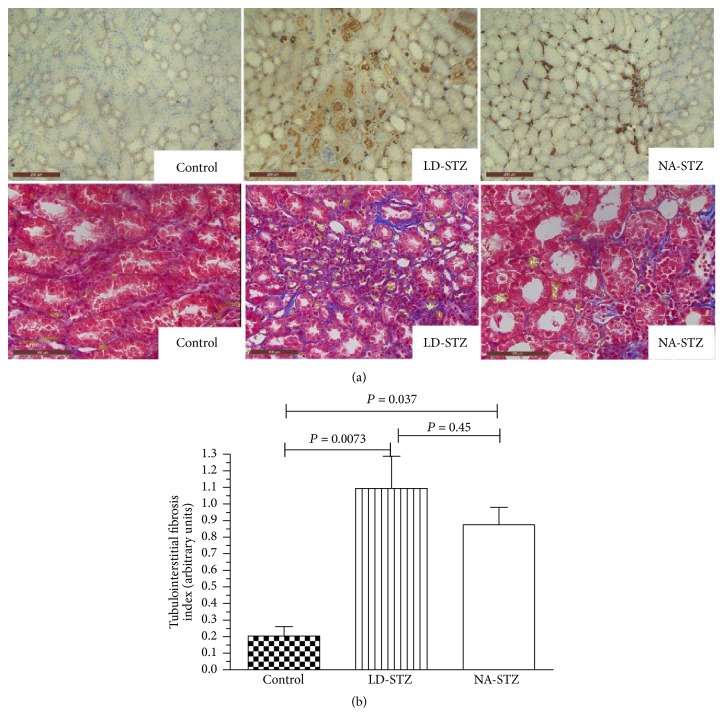
Tubulointerstitial lesions in experimental groups at week 30. (a) Representative immunostaining with anti-collagen type IV antibodies (upper row, original magnification ×200) and Masson's trichrome staining (lower row, original magnification ×320) of kidney sections from control nondiabetic group, low-dose streptozotocin-injected group, and nicotinamide-streptozotocin-induced diabetic group. Kidney tissues from both diabetic groups demonstrate tubulointerstitial lesions with tubular dilation and partial tubular atrophy compared to control nondiabetic rats and are characterized by interstitial inflammatory infiltration (indicated by white arrows on Figure 11(a)). Marked increase expression of collagen type IV is seen in tubular basement membrane, vascular walls, and interstitial areas in both diabetic groups. (b) Graphic representation of semiquantitative indices of tubulointerstitial fibrosis in control and two diabetic groups. Mean values and standard deviations are shown.

Semiquantitatively evaluated glomerulosclerosis and tubulointerstitial fibrosis indices (Figures 11 and 12, resp.) were significantly higher in diabetic groups compared to nondiabetic animals. However, there were no significant differences in both indices between LD-STZ and NA-STZ-injected diabetic rats.

**Figure 13:**
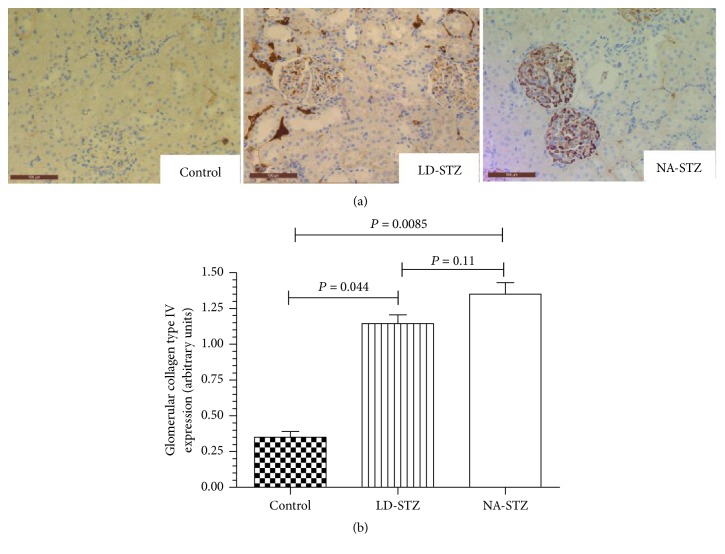
Immunostaining of kidney cortex sections from control and diabetic rats with anti-type IV collagen antibodies. (a) Representative microphotographs from the experimental groups (magnification ×320). The markedly increased immunostaining is present in glomerular basement membranes and mesangial matrix of both diabetic groups as compared to control nondiabetic rats. (b) Comparison of the quantified staining intensity in glomeruli of the studied groups. Expression of type IV collagen was assessed semiquantitatively. Mean values and standard deviations are shown.

The immunohistochemical staining for type IV collagen in the glomerular area of both diabetic groups showed darker brown pigmentation compared to the control group. However, significant difference between LD-STZ and NA-STZ-injected diabetic rats was not observed as well (Figure 13).

## 4. Discussion

According to the data posted by Nephropathy committee on The US-based Animal Models of Diabetes Complications Consortium website (AMDCC, https://diacomp.org/), strict morphological and laboratory validation criteria are offered for an ideal rodent model of DN. They are 50-fold increase in albuminuria compared to the control, decline in creatinine clearance more than 50% from initial level during follow-up period, at least twice-thicker GBM compared to initial, and the presence of mesangial sclerosis, arteriolar hyalinosis, and tubulointerstitial fibrosis. Thus, tubulointerstitial injury has to be evaluated along with the glomerular disturbances. It is established that in most cases tubulointerstitial injury determines rather progression of DN than its onset [4, 21]. However, recent studies have shown that elevation of tubular injury markers could occur very early in the settings of DM2 and metabolic disorders, prior to the development of evident DN [31, 32, 40].

The main idea of our study was to compare the features of DN in two rat models with similar degrees of metabolic disorders. To create NA-STZ-induced model of DM2 with DN, we decided to modify the technique of Islam and Choi [41] by complementation with unilateral right nephrectomy and the five-week-high-fat diet with beef tallow. This diet was previously described by Logan as an independent factor for glucose intolerance and renal disturbance progression [14]. To compare our model with a single low-dose-STZ-induced DM2, we adapted the translational model of DM2 with DN presented by Sugano et al. [16]. However, some validation criteria of an appropriate model of DN including tubulointerstitial injury and GBM thickening were not evaluated in this model.

In our study obtained deterioration of renal morphofunctional changes in NA-STZ group was confirmed by almost 40-fold increase of urinary albumin excretion rate as compared to the nondiabetic group and gradual moderate decline in creatinine clearance throughout the observation period. Furthermore, early ultrastructural features specific for diabetes (significant glomerular basement membrane thickening and partial podocyte foot process effacement) have been observed by week 20 and progressed by the end of the experiment. Additionally, more advanced histological findings by week 30 (such as significantly expanded mesangium and the presence of sclerosis in glomeruli and tubulointerstitium) show certain significant similarities to the classical natural history of human diabetic kidney disease [3, 4].

As compared to LD-STZ group, by week 10 of the experiment, animals in both diabetic groups developed the same important features of metabolic syndrome. However, all of them seemed to be more stable until the end of the study only in NA-STZ group. Regarding renal morphofunctional disturbances, significant differences were detected only in terms of albuminuria and mesangial expansion (both were more pronounced in NA-STZ group). Nevertheless, a significantly lower value of NGAL was obtained in NA-STZ group as compared to LD-STZ group at weeks 10 and 30. It could indicate that even the low-dose STZ administration could have early as well as long-term effects on tubular function. However, there have not been observed significant differences in the degree of renal tubulointerstitial lesion, although it was weaker in NA-STZ group compared to LD-STZ. It might be suggested that nephrotoxic effects of STZ were less pronounced in the group of rats receiving NA prior to a high dose of STZ. Interestingly, other light microscopic features were less pronounced in LD-STZ group. Therefore, it seemed to be surprising that the deterioration of low-dose-STZ-mediated tubular dysfunction was not associated with the progression of DN.

Based on all of these observations, we suggested several speculations about them. Firstly, as metabolic disorders were very similar in both diabetic groups at week 10, observed difference in the degree of urinary tubular injury marker (NGAL) could be attributed, at least partially, to the influence of preliminary injection of NA. In spite of this, even in case of using NA together with STZ to induce DM2, we recommend to start assessing any morphofunctional specimens from experimental animals not earlier than 2 weeks after STZ administration. According to the published data, assessment of pharmacological effects on the development of DN should not be started until at least 3 weeks after STZ use to avoid STZ-mediated renal artefacts [6, 8]. Secondly, even if a significant increase in the levels of kidney injury markers in LD-STZ group at week 10 is associated with the toxic effects of STZ on tubular cells, most likely this influence can be ignored due to very similar basic diabetes-related renal morphofunctional changes. Also it could be speculated that summative effect of continuous metabolic disturbances has greater impact on the development of long-term renal changes than early LD-STZ-mediated tubular dysfunction does. Thus, our study has extended the notion of differences between these two models of DN in DM2 from the standpoint of some glomerular disturbances as well as tubular function.

Proposed model of NA-STZ-induced DM2 with DN was successfully used in our previous pilot study to investigate the renal effects of metformin, where the drug demonstrated nephroprotection regarding tubular function beyond glucose lowering properties [42]. Interestingly, a similar effect of metformin on urinary renal injury markers was obtained in our clinical study [43]. Meanwhile, it was not detected when modelling STZ-induced DM in rats [44], described by Zhang et al. [17]. They proposed a model of DM2 that was induced in male Wistar rats by monthly high-fat diet (commercial high-fat chow) and two intraperitoneal injections of a low dose of STZ (30 mg/kg) in one-week interval [17]. However, in our experience, two days after the second injection of STZ, the rats developed severe hyperglycemia [44]. Similar results in regard to glycaemia were described in another pilot study using STZ in a dose of 60 mg/kg in high-fat-fed rats [45]. Thus, neither low dose of STZ administered twice nor single higher dose of STZ following the short period (up to 5 weeks) of feeding with beef tallow could induce metabolic disorders typical for experimental DM2 [33].

The present study has some limitations. Thus, we did not use the group with a single high-dose-STZ-induced DM (65 mg/kg) as a more appropriate control to NA-STZ group to confirm tubuloprotective properties of preliminary NA administration. We suppose that it is quite difficult to compare early tubular effects using only urinary injury markers in these models, since significant differences in the levels of glycaemia between the groups would occur [6, 45]. This affirmation is based on the knowledge that hyperglycemia per se could injure tubulointerstitium [4, 21]. Moreover, several clinical studies have indicated high correlation between KIM-1 and NGAL urinary excretion and degree of diabetes compensation [31, 32, 46]. Second limitation is related to the absence of data on ultrastructural renal changes in LD-STZ group at both time points because of the technical issues described above. Another limitation is that we did not measure blood pressure, because arterial hypertension is not an obligate feature for the validation of DN model as was mentioned above. Finally, less stable metabolic disorders observed in LD-STZ group might be related, at least partly, to the usage of five-week-high-fat diet instead of the continuous feeding originally described in the study of Sugano et al. [16]. As a consequence, to increase the reproducibility of metabolic disorders, we recommend continuing the high-fat feeding at least up to 2 months. According to the data obtained by Logan, the usage of high-fat diet of this duration allows us to model impaired glucose tolerance, insulin resistance, and glomerular disturbance without any additional interventions [14].

## 5. Conclusions

We proposed a new model of type 2 diabetes with diabetic nephropathy characterized by stable metabolic disorders, typical renal lesions, and lower degree of tubular dysfunction as compared to the low-dose streptozotocin-induced diabetes.

Described combined nicotinamide and streptozotocin usage in uninephrectomized high-fat-fed rats seems to be more suitable for the investigation of early stages of diabetic nephropathy as compared to the low dose of streptozotocin administration when renal tubular function is considered. Nevertheless, it still remains unclear which approach to diabetic nephropathy modelling is the most appropriate when only classical renal diabetic morphofunctional features are evaluated.

## Supplementary Material

Under anesthesia after shaving and cleansing, right vertical extra-peritoneal lumbar incision was performed, the right renal pedicle was ligated at two points. To preserve adrenal gland against extraction, circular incision of renal capsule was performed, and kidney after separate enucleation was removed. The incision was closed with simple continuous suture. The skin was closed with simple intermittent sutures.

## Figures and Tables

**Table 1:** Summary of metabolic parameters and serum levels of urea and creatinine in the control, NA-STZ, and LD-STZ groups measured at 10, 20, and 30 weeks of the experiment. Mean values and standard deviations are shown. *P*  versus NA-STZ: comparison of nicotinamide-streptozotocin-induced diabetic rats and the other two experimental groups at the same point of measurement. *P*  versus LD-STZ: comparison of low-dose-streptozotocin-induced diabetic rats and the other two experimental groups at the same point of measurement. N/A: not assayed.

Experimental groups	Control nondiabetic group			Low-dose streptozotocin-induced diabetic group			Nicotinamide-streptozotocin-induced diabetic group		
Parameters Mean ± standard deviation	Week of the study			Week of the study			Week of the study		
	10 weeks	20 weeks	30 weeks	10 weeks	20 weeks	30 weeks	10 weeks	20 weeks	30 weeks
HbA1 c, %	3.6	3.7	4.0	5.45	6.85	6.7	5.39	7.02	6.94
SD	0.29	0.13	0.26	0.61	0.21	0.43	0.34	0.29	0.27
*P* versus NA-STZ	0.029	0.01	0.016	0.97	0.39	0.46			
*P* versus LD-STZ	0.028	0.029	0.028						

Serum insulin level, pmol/L	83.5	96.6	87.8	50.2	54.0	59.6	49.6	51.3	50.9
SD	6.81	9.18	6.24	8.13	10.46	8.93	7.34	8.72	8.85
*P* versus NA-STZ	0.028	0.009	0.015	0.88	0.76	0.27			
*P* versus LD-STZ	0.03	0.027	0.28						

HOMA-IR	1.93	2.23	2.01	2.76	2.78	2.83	3.20	2.87	2.91
SD	0.29	0.14	0.22	0.92	0.93	0.87	0.39	0.22	0.70
*P* versus NA-STZ	0.028	0.014	0.031	0.31	0.99	0.77			
*P* versus LD-STZ	0.11	0.48	0.06						

Total cholesterol, mmol/L	1.55	1.58	1.63	2.83	2.71	2.42	2.89	2.76	2.74
SD	0.35	0.20	0.27	0.35	0.19	0.32	0.25	0.23	0.21
*P* versus NA-STZ	0.028	0.014	0.002	0.88	0.74	0.11			
*P* versus LD-STZ	0.028	0.031	0.008						

Triglycerides, mmol/L	0.57	0.56	0.70	1.13	1.04	0.86	1.05	0.90	0.89
SD	0.08	0.12	0.17	0.49	0.41	0.33	0.18	0.12	0.19
*P* versus NA-STZ	0.028	0.01	0.029	1.0	0.91	0.98			
*P* versus LD-STZ	0.029	0.057	0.21						

Serum urea, mmol/L	N/A	N/A	6.5	N/A	N/A	8.7	N/A	N/A	8.82
SD			0.89			1.18			1.03
*P* versus NA-STZ			0.06			0.96			
*P* versus LD-STZ			0.012						

Serum creatinine, *μ*mol/L	33.5	35.8	41.9	41.3	44.8	61.5	38.3	47.0	69.0
SD	3.42	3.61	4.09	6.24	6.50	11.62	2.99	4.0	4.30
*P* versus NA-STZ	0.14	0.004	0.002	0.65	0.80	0.31			
*P* versus LD-STZ	0.11	0.038	0.008						
